# Remembering through the genome: the role of chromatin states in brain functions and diseases

**DOI:** 10.1038/s41398-023-02415-4

**Published:** 2023-04-11

**Authors:** Rodrigo G. Arzate-Mejia, Isabelle M. Mansuy

**Affiliations:** 1 Laboratory of Neuroepigenetics, Brain Research Institute, Medical Faculty, University of Zurich, Zurich, Switzerland; 2 Institute for Neuroscience, Department of Health Science and Technology, Swiss Federal Institute of Technology Zürich (ETHZ), Zurich, Switzerland; 3 Center for Neuroscience Zürich, University Zürich and ETHZ, Zürich, Switzerland; 4 Present Address: Center for Neuroscience Zürich, University Zürich and ETHZ, Zürich, Switzerland

**Keywords:** Neuroscience, Epigenetics and behaviour

## Abstract

Chromatin is the physical substrate of the genome that carries the DNA sequence and ensures its proper functions and regulation in the cell nucleus. While a lot is known about the dynamics of chromatin during programmed cellular processes such as development, the role of chromatin in experience-dependent functions remains not well defined. Accumulating evidence suggests that in brain cells, environmental stimuli can trigger long-lasting changes in chromatin structure and tri-dimensional (3D) organization that can influence future transcriptional programs. This review describes recent findings suggesting that chromatin plays an important role in cellular memory, particularly in the maintenance of traces of prior activity in the brain. Inspired by findings in immune and epithelial cells, we discuss the underlying mechanisms and the implications for experience-dependent transcriptional regulation in health and disease. We conclude by presenting a holistic view of chromatin as potential molecular substrate for the integration and assimilation of environmental information that may constitute a conceptual basis for future research.

## Introduction

### Experience-dependent changes in the brain

The mammalian brain is a complex organ whose functions are strongly influenced by experience. Across life, the brain needs to assimilate and integrate information about the environment, and use this information to adapt and direct actions and behavior. These biological functions are fundamental for animals and human beings and have a social, ecological and evolutionary impact [1–3]. Early life experiences can influence the establishment of cellular states that shape brain functions in adulthood. For instance, adversity during the first weeks or months of life can alter cognitive and behavioral responses in adulthood and is a risk factor for neurodevelopmental and psychiatric disorders [4, 5]. In contrast, positive experiences involving for instance, enriched social settings can have durable beneficial effects on behavior [6]. Mechanistically, the way life experiences affect the brain on the long-term is known to involve cellular plasticity and stability. Neuronal and glial cells that constitute and regulate synaptic circuits and drive activity-dependent responses have properties of plasticity and stability [7]. But how this is controlled at the molecular level remains not well defined. In recent years, accumulating evidence suggested that experience can persistently modify chromatin composition [8, 9], structure [10–13] and 3D organization [14–19]. Chromatin modifications have been proposed to influence transcriptional activity and modulate brain cells functions [20, 21]. They may therefore contribute to the etiology of brain pathologies and the emergence of neurodevelopmental and psychiatry disorders [22–25].

### Chromatin organization and functions

In the cell nucleus, genome organization relies on chromosomal interactions and on associations between chromatin and the nuclear lamina at the periphery of the nucleus [26, 27]. Chromatin is composed of the DNA, our genetic material, that is associated with proteins and RNA. Chromatin is packed into chromosomes and has a hierarchical organization starting with nucleosomes formed by DNA and histone proteins [28]. Nucleosomes can interact with each other via histones and their covalent post-translational modifications, such as acetylation, methylation, phosphorylation, and ribosylation [29–32]. Histone modifications are established by an ensemble of enzymes called “writers,” “readers,” and “erasers” that add, decode, or remove modifications, respectively. These proteins are essential for brain development and functions and their dysregulation has been associated with brain diseases [33, 34]. Depending on the nature of their modifications, histones can have different electrostatic properties and be repulsive or attractive to each other. This consequentially establishes regions of open and accessible chromatin called euchromatin, and regions of compacted and less accessible chromatin called heterochromatin. Regions of open chromatin can more favorably bind proteins, such as transcription factors (TFs) and regulators [31, 32]. Together with ATP-dependent chromatin remodeling complexes, TFs bound to DNA can displace nucleosomes to create further regions of accessibility [32, 35, 36]. These TF-binding regions are generally regulatory elements such as promoters, enhancers, silencers and insulators [37, 38]. An important property of chromatin is that it can adopt a bivalent state characterized by the co-occurrence of histone modifications associated with gene activation such as histone H3 Lys4 trimethylation (H3K4me3) and histone modifications associated with gene repression (H3K27me3), for instance at the promoter of developmental genes during cell-lineage specification [39]. Such bivalent state is thought to poise important regulatory genes or regions for expression or repression.

Regulatory elements can physically interact via long-range interactions and create chromatin loops [40]. At a high level of organization, groups of chromatin loops can be located within self-interacting regions called topologically-associating domains (TADs). Chromatin can also segregate into active and inactive compartments by preferential physical interaction of regions with similar biochemical composition. Regions with active transcription and histone modifications associated with open chromatin generally segregate into active compartments (A) while regions with repressive histone modifications segregate into inactive compartments (B) [26, 28, 40].

The 3D organization of the genome is an essential component of transcriptional regulation in mammals and undergoes major changes during development and cellular activity [37, 41, 42]. Short- and long-range physical interactions can form between *cis*-regulatory elements, particularly enhancers and promoters, and modulate gene transcription, for instance in differentiating stem cells, during circadian cycle and even during activation of brain cells [43–45]. Chromatin long-range interactions between regulatory elements can also fine-tune the specificity and timing of activity-dependent gene expression [13, 14, 16, 19, 46]. In the brain, neuronal activity induces fast interaction between the enhancer and promoter of the immediate-early genes (IEGs) *c-Fos* and *Arc*, which leads to gene transcription [14, 47]. Importantly, chromatin loops can form before transcription and thereby, create a primed state that facilitates rapid and specific future transcriptional responses [48–50].

Mechanistically, the establishment and stabilization of long-range interactions and chromatin loops primarily involve the activity of the architectural proteins cohesin and binding of the CCCTC-binding factor (CTCF) [51, 52]. They are also influenced by covalent modifications of the DNA and histone proteins at interacting regulatory sequences and by the binding of TFs and co-activators/repressors such as Mediator and Polycomb repressor complexes [31, 53–55]. These factors are cell-type specific and stimulus-dependent, and thus vary depending on the tissue and type of exposure. Chromatin loops can be formed by either an active process of loop extrusion by cohesin and CTCF, or a process of phase separation of genomic regions with similar histone modifications that tend to naturally interact [26, 28]. For regulatory long-range interactions involving enhancers and promoters, the repertoire of histone modifications and binding partners determines if transcription of nearby sequences is favored or repressed [56]. When activated, enhancers are bidirectionally transcribed into enhancer RNAs and their flanking nucleosomes are typically marked by H3K27 acetylation (H3K27ac) and H3K4 methylation (H3K4me1) [57, 58]. Enhancers can be poised and lack H3K27ac but possess H3K4me1 and Polycomb complexes marked by H3K27me3. Poised enhancers are transcriptionally silent but can be activated in a time- and lineage-specific manner, for instance in embryonic stem cells during differentiation [59]. Enhancers can also interact with long non-coding RNAs via cohesin-CTCF, repressive complexes or epigenetic modifiers such as the histone acetyltransferases p300 and CREB-binding protein [60–62].

### Role of chromatin in the brain

Chromatin-based mechanisms that control gene expression contribute to cell differentiation during brain development and to higher-order functions in the adult. Early postnatal life is a time of intense epigenome remodeling in brain cells. In the maturing brain, enhancer–promoter interactions undergo global rewiring, and TADs and chromosomal compartments are reorganized within neuronal and glial cells nuclei [63, 64]. At the level of the epigenome, regulatory elements such as enhancers are extensively remodeled at the level of histone modifications. For instance, H3K27ac is dynamically regulated during the first 3 weeks of postnatal life, which coincides with the timing of changes in gene expression [65]. Importantly, enhancers that are activated or repressed already in the postnatal brain are maintained in their respective activated or repressed state throughout adulthood [65].

Chromatin states are also modified by neuronal activity, which involves activation of enhancers [9]. Several enzymes involved in epigenetic processes including CREB-binding protein, chromatin remodelers such as BRG1/BRM-associated factor and the nucleosome remodeling deacetylase NuRD are associated with gene activation or repression in neurons [66–69]. Mechanistically, the regulation of chromatin structure and 3D organization differs depending on the brain region and type of stimulation. For instance, while activity during cortical maturation modifies chromatin conformation in the visual cortex, sensory deprivation does not [15, 70]. Functionally, changes in 3D chromatin organization in the adult brain are associated with key processes such as learning and memory. Motor learning induces long-range interactions between enhancer-promoter elements and transcriptionally active compartments in cerebellar granule neurons, which results in gene expression [18]. Likewise, remote memory storage in the brain correlates with enhancer-promoter activation and persistent transcriptional and chromatin changes in several neuronal and glial cell populations in the adult hippocampus and prefrontal cortex [10, 71].

Overall, changes in chromatin structure and 3D organization have been associated with neurodevelopmental processes and higher-order brain functions in adulthood, but emerging evidence suggests that they can also be implicated in experience-dependent regulatory memory in brain cells. Below we review current evidence supporting a role for chromatin in the maintenance of traces of prior activity in brain cells and we describe potential mechanisms of establishment and functional implications in health and disease.

## Neuronal activation can induce stable changes in chromatin structure and 3D organization

In mammals, neuronal activation induces the expression of genes necessary for neuronal plasticity, a cellular property essential for learning, memory and cognition [72]. At the transcriptional level, neuronal activation is characterized by the induction of two successive waves of transcription: (1) a first wave during which IEGs coding for TFs and epigenetic modifiers are transcriptionally activated or repressed within minutes after membrane depolarization, (2) a second wave characterized by the expression of genes activated by IEGs involved in neuronal plasticity [73]. IEGs transcription is rapidly and transiently induced, and returns to pre-stimulation level within a couple of hours [13, 72, 73]. Such stereotypic transcriptional response is accompanied by changes in chromatin structure and 3D organization [13, 14]. In particular, TFs activation leads to the recruitment of transcriptional co-factors and RNA Pol II at enhancers and promoters, which increases chromatin accessibility at thousands of genomic sites and reconfigures the 3D genome.

Unlike transcription, changes in the structure, composition or 3D organization of chromatin that occur after IEGs activation are not transient and chromatin is not necessarily re-established at pre-stimulation states. For instance, neuronal activation by electroconvulsive stimulation increases chromatin accessibility at thousands of genomic sites after one hour in hippocampal slices [74]. But although chromatin seems to overall progressively return to a pre-stimulation state, hundreds of sites remain accessible for at least 24 h. Although most of the persistent sites are located upstream transcription start sites (TSS), genes in these regions are no longer differentially expressed, suggesting that retention of chromatin accessibility at these sites is not anymore linked to differential gene transcription. Further, while the activity-dependent TF c-Fos occupies persistent sites upon neuronal activation, it is no longer present at these sites 24 h later [74] and may be replaced by constitutive and/or cell-type specific TFs (see Mechanisms).

Kainic acid, frequently used to induce seizures in animal models, also persistently affects chromatin in neurons. A single injection is sufficient to strongly activate neurons and increase transcription in excitatory neurons of the adult hippocampus. It extensively changes chromatin accessibility and long-range 3D interactions between regulatory elements [13]. Like with electroconvulsion, thousands of genomic sites retain chromatin accessibility, in this case up to 48 hours after kainic acid treatment. These lasting changes contrast with transient transcriptomic changes that largely return to basal states. This supports the idea that chromatin accessibility is not directly driven by transcription per se or associated with emergent transcriptional programs. Further, even when transcription returns to basal levels, TFs can remain bound and RNA polymerase can stay in a paused state. This can allow easier initiation of transcription at a later timepoint, which then only requires release of the paused transcription complex. This can also constitute a form of chromatin memory [75, 76].

Remarkably, genomic regions with lasting changes in chromatin accessibility are also engaged in persistent long-range interactions that are independent of CTCF binding. These regions are enriched for the binding motif of the family of TFs AP-1, suggesting a role for AP-1 TFs in the establishment and maintenance of chromatin states (see Mechanisms). Sites with stable changes in occupancy are also located at the promoter or enhancer of genes involved in axonal growth or protein aggregation [13], suggesting that they may influence future transcriptional responses by acting as chromatin priming events.

## Salient experiences lead to stable changes in chromatin structure and composition

Environmental exposure and salient experiences, i.e., experiences that stand out, are extreme and emotionally charged, can have long-lasting effects on chromatin in brain cells. Their effects can vary depending on the time window of exposure (whether before or after birth or in adulthood), but also its type (e.g., stress, drug of abuse) and duration (acute or chronic). While the adult brain is susceptible to changes, the developing brain is probably even more sensitive and may be more profoundly affected since many developmental processes such as cell division and differentiation in prenatal life and cell migration, synaptogenesis and myelination after birth are ongoing [77]. Further to their salience, the valence of experiences (whether perceived as positive or negative) also matters and events of equal salience but differing valence may affect chromatin differently.

### How early life exposure affects chromatin states

Early life conditions such as stress before or after birth can have effects on physiology, behavior and cognition that can last until adulthood [78]. These effects have been associated with transcriptional and epigenetic changes in components of the hypothalamic–pituitary–adrenal (HPA) axis, neurotransmitter signaling and neuroimmune pathways in neuronal and glial cells in the brain [79–83]. For instance, chronic stress between postnatal day (PND) 10 and 17 in mice globally alters the level of H3K79me1/2 in nucleus accumbens (NAc) in the adult brain, a region involved in motivation and reward [12]. This effect is linked to transcriptional dysregulation of *Dot1l* and *Kdm2b*, which are H3K79 methyltransferase and demethylase respectively, in dopaminergic neurons of NAc [11]. Although chromatin itself was not examined, it is possible that its structure is affected since H3K79me2 can alter nucleosome surface [84] and enhance accessibility to TFs [85]. Since *Dot1l* dysregulation occurs only in early adulthood, the observed changes in H3K79me1/2 are not an immediate consequence of stress at the time of exposure but rather emerge later in development, suggesting a form of molecular memory of prior stress.

While transcriptional and epigenetic changes have been well studied in animal models and humans, changes at the level of chromatin itself remain not well characterized in the brain. Most evidence for changes in chromatin by stress is in immune cells. Perinatal glucocorticoids have been shown to favor chromatin accessibility in cytotoxic T cells at genes carrying glucocorticoid receptor binding sites necessary for interferon production [86]. The effects are in part due to negative feedback pathways mediated by mineralocorticoid receptor signaling in the hippocampus and hypothalamus, which result in an overall decrease in circulating corticosterone [87]. Both mineralocorticoid and glucocorticoid receptor signaling can enhance chromatin accessibility [88, 89] and modify chromatin composition by promoting the recruitment of chromatin remodeling enzymes, co-factors, and other TFs.

Further to stress, exposure to chemicals early in life can also affect behavior and cognition in mammals and involve lasting changes in chromatin structure and organization affecting gene expression in the brain. Repeated exposure to midazolam, an anesthetic frequently used in children, can cause neurological abnormalities. In mouse, midazolam exposure from PND10 to PND12 impairs hippocampus-dependent object memory on a novel location recognition task as well as fear memory on a contextual fear conditioning test [90]. At the cellular level, this is accompanied by reduced proliferation and differentiation of neuronal stem cells (NSCs), leading to decreased cellular thickness of the dentate gyrus. The cognitive and cellular effects could be related to long-lasting changes in chromatin accessibility in NSCs. Thousands of genomic sites have increased chromatin accessibility after midazolam exposure at PND10 and some of them retain accessibility in adult NSCs (8 weeks old), suggesting persistent changes in chromatin states. The sites are particularly enriched at the promoter of genes involved in quiescence, and genes transcriptionally induced by midazolam that remain activated in adult NSCs. Notably, the defects in proliferation, differentiation and cognition can be partially reversed by voluntary exercise in adult mice [90], although gene expression is only modestly restored, suggesting that changes in chromatin accessibility may themselves have persisted.

### Evidence of changes in chromatin states by exposure in adulthood

Memory formation has been associated with long-lasting changes in chromatin accessibility. Neuronal activation by fear conditioning rapidly increases chromatin accessibility at thousands of genomic loci in hippocampal neurons (within 1.5 h). Some of the changes persist for at least five days, suggesting lasting chromatin states [10]. Conditioning also persistently affects chromatin 3D organization at hundreds of genomic regions (with an average size of 400 kb). Many of these regions switch from compartment B (inactive) to compartment A (active) after conditioning and half of those that remain in compartment A are associated with long-lasting changes in chromatin accessibility. Genomic sites with lasting changes have three important features: (1) They are enriched in histone post-translational modifications such as H3K4me1 and H3K4me1/H3K27ac that are associated with primed and active enhancers respectively, (2) they are enriched in binding motifs of activity-dependent TFs such as AP-1 and architectural proteins such as CTCF and YY1, and (3) they engage in constitutive or dynamic long-range interactions with other regulatory elements during memory formation and recall. Remarkably, upon memory recall, some of the open genomic sites have more frequent physical interaction with their cognate promoters or engage in novel long-range interactions, resulting in transcriptional activation of their target genes [10]. This suggests that a subset of genomic sites that can maintain chromatin states is functional and can affect gene transcription upon recall.

Further to memory, addiction has also been associated with lasting changes in chromatin. Addiction is a pathological dysfunction characterized by a loss of self-control over one’s action that can be harmful. One of the detrimental features associated with addiction is relapse, that can occur even after prolonged abstinence and is caused by “drug” memory. In mice, chronic exposure to cocaine followed by 30-day withdrawal increases chromatin accessibility at thousands of genomic regions including potential enhancer elements, in medium spiny neurons of NAc expressing the D1 dopamine receptor (cocaine target) [11]. Some of these regions are located close to genes stimulated by the drug. For instance, *ΔFosB* promoter and enhancer elements have increased chromatin accessibility upon acute cocaine administration that persists during prolonged withdrawal and upon relapse [11]. Importantly, genes located nearby regions with increased accessibility have upregulated transcription after relapse when compared to transcription after acute treatment or during withdrawal following chronic cocaine administration. This suggests that chronic cocaine can induce a form of chromatin priming that influences subsequent transcriptional responses upon re-exposure. Whether the primed regions are causally involved in transcriptional activation is however not determined.

Further to neuronal cells, immune cells in the adult brain can also acquire lasting changes in chromatin composition. In adult mice, peripheral inflammation induced by lipopolysaccharides injection causes differential deposition of H3K4me1 and H3K27ac at enhancer elements that persist for at least 6 months in microglia, brain resident macrophages [91]. Enhancers with lasting changes in histone marks are associated with genes involved in signaling such as thyroid hormone pathway and hypoxia inducible factor-1α pathway. Since the presence of histone marks such as H3K4me2 is closely associated with regions of chromatin accessibility in microglia [92, 93], the data suggest that inflammation may result in a form of inflammatory memory via long-lasting effects on chromatin accessibility.

## Potential molecular mechanisms for the establishment and maintenance of chromatin changes in the brain

### Transcription factors

**Fig. 1:**
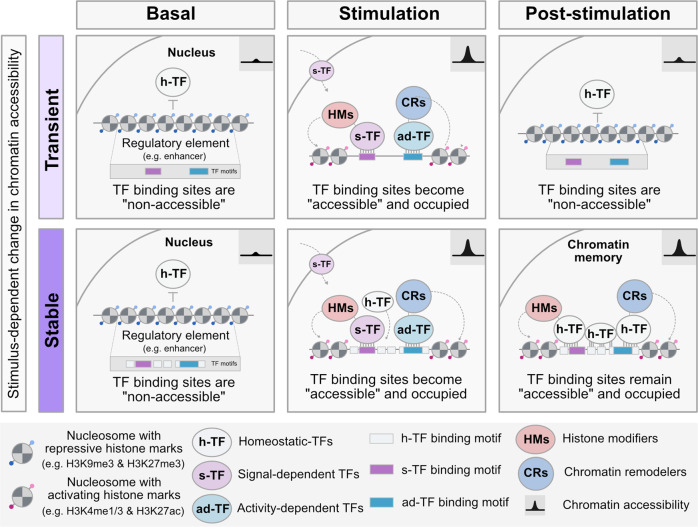
TF-dependent establishment and maintenance of chromatin memory. Model illustrating a TF-dependent mechanism for the establishment of transient (top) or stable (bottom) changes in chromatin accessibility at regulatory elements (inspired by findings reported in [10, 13, 74, 95, 97, 100, 101, 104, 121–123]. Left: in basal state, nucleosomes with repressive histone modifications, i.e., H3K9me3 and H3K27me3 (and with DNA methylation, not depicted in the figure) at a regulatory element, i.e., enhancer, represent a barrier for TF binding via occlusion of binding motifs [95, 122, 123]. Homeostatic TFs (h-TF) cannot stably bind nor initiate chromatin remodeling to displace nucleosomes, regardless of the underlying DNA sequence (top or bottom). Middle: Upon stimulation such as neuronal activation or inflammation, a type of TFs termed signal-dependent (s-TF), i.e., STAT3, present at the nuclear membrane is activated [101] then translocates to the nucleus where it binds to DNA binding motifs located at regulatory elements in a chromatin context otherwise non-accessible to h-TF [101, 124, 125]. This is accompanied by the recruitment of chromatin remodeling (CR) complexes that induce nucleosome mobilization and histone modifications which results in the exposure of additional TF binding motifs making the regulatory element “accessible” [10, 13, 126]. s-TFs induce the transcription of another class of TFs, termed activity-dependent (ad-TF), i.e., AP-1 class such as c-Fos or Egr1, which can be recruited to newly accessible regulatory elements and activate these elements by recruiting RNA Pol II and DNA demethylases [13, 101]. Regulatory elements with increased chromatin accessibility can also be occupied by h-TF. In this scenario, h-TF binding depends on the sequence of the regulatory element and its accessibility determined by epigenetic modifiers recruited by s-TF and ad-TF. The transient versus stable state is determined by the nature and number of TF binding motifs in the regulatory element. Right: After stimulation, the majority of responsive regulatory regions return to a basal state of chromatin accessibility (Transient). Responsive regions with chromatin memory have persistent changes in chromatin accessibility, here shown as a gain in accessibility resulting from h-TF binding (Stable). Depicted histone modifications are only those mentioned in the text, many other modifications exist but are not drawn for simplicity. Additional mechanisms may also contribute to the formation of chromatin memory in the brain.

One of the consequences of the genomic redistribution of DNA binding proteins such as TFs are changes in chromatin accessibility. Because TFs can remain bound to DNA, they have been proposed to regulate the establishment and maintenance of chromatin states [94–96]. Consistently, genomic regions with lasting increase in chromatin accessibility after midazolam treatment are enriched in binding motifs for the architectural protein CTCF but also for TFs of Egr1 and Nfi family [90]. But while Egr1 expression is transient, its target regions remain open for almost 60 days after midazolam exposure in adult NSCs. This paradoxical phenomenon may be explained by a mechanism involving at least two phases. In the first phase, an environmental signal induces the transient activation of specific TFs i.e. signal-dependent and activity-dependent TFs, and epigenetic modifiers, i.e., DNA methylases and de-methylases or chromatin remodelers. This results in the remodeling of chromatin at target regulatory elements, an event known as hit-and-run due to the action of transiently induced TFs [94–100]. In the second phase, constitutively expressed TFs called homeostatic TFs, maintain the chromatin status of targeted regulatory elements by binding to exposed DNA-binding motifs (Fig. 1) [95, 96, 100, 101]. Since Egr1, an activity-dependent TF, physically interacts with chromatin remodelers and epigenetic modifiers such as TET enzymes [102, 103], it may favor the binding of other TFs next to Egr1-binding sites. This can result in the active de-methylation and modification of chromatin structure, as suggested during postnatal maturation of neurons in mice [102]. In postnatal neurons, Egr1 binding sites themselves have been found to be demethylated [102]. With such scenario, the binding of additional TFs may help maintain the new chromatin configuration at these sites even in the absence of Egr1. Because Egr1 sites can be co-occupied by other TFs of the Nfi family and by chromatin remodelers such as Chd8 and Smarca4 in NSCs, these proteins have been suggested to help maintain chromatin accessibility [90]. However, since they are already present at Egr1 binding sites in wild-type NSCs, it is not clear how they can further act. Perhaps other regulatory proteins, possibly different members of the Nfi TFs family may help maintain chromatin states at Egr1 sites.

An epigenetic mechanism for dynamic changes in gene expression mediated by lasting modifications of chromatin accessibility has also been proposed in the context of neuronal activation [74]. Increased c-Fos expression and binding associated with chromatin accessibility induced by activity are only transient and disappear 4 h after neuronal activation. Since chromatin itself remains open, it is proposed that other binding factors of the same family replace c-Fos after it is freed from its genomic sites [74].

While still limited in brain cells, there is evidence that TFs play a role in the establishment, maintenance and reactivation of chromatin states in the context of inflammatory memory in adult stem cells [101, 104]. In epidermal stem cells, a local inflammatory challenge changes chromatin accessibility for up to 6 months. The changes involve the signal-dependent TF STAT3 that promotes the expression of the activity-dependent TF c-Fos and its joint recruitment with the constitutive TF Jun to enhancers and promoters [101]. But while c-Fos binding to chromatin is only transient, Jun and other constitutive TFs like ATF3, also member of AP-1 family, and p63, remain bound [101]. This suggests that once a genomic region becomes accessible due to binding of activity-dependent TFs, it can be populated by additional TFs given the presence of their DNA binding motif. (Fig. 1). This also suggests that regulatory elements with the potential to exhibit chromatin memory have binding motifs for induced and constitutive TFs. Consistently, bioinformatic characterization of sequence features of regions with prolonged chromatin accessibility reveal high enrichment of binding motifs for AP-1 family TFs. These TFs have similar DNA binding motifs and are both constitutive and activity-dependent, suggesting that the presence of multiple AP-1 motifs may be a feature of regions with “chromatin memory.” Importantly, the presence of multiple independent binding sites for AP-1 TFs in regions with lasting changes in chromatin accessibility could confer resilience to alterations in the availability of TFs that occupy regions with chromatin memory. In support of this, the removal of Jun does not affect chromatin accessibility at regions with chromatin memory nor the recruitment of other TFs such as ATF3 [101]. This suggests that the binding of these TFs is independent and provides robustness to regions with chromatin memory. However, whether the loss of Jun alters the functionality of such regions remains to be determined. But overall, these data support a hit-and-run mechanism for the establishment of chromatin memory and a role for TFs normally expressed in the cell type of interest in the maintenance of long-lasting chromatin accessibility at regulatory elements (Fig. 1).

A hit-and-run mechanism mediated by constitutive and environmentally induced TFs has also been described in adult hematopoietic stem cells to explain long-lasting changes in chromatin accessibility induced by lipopolysaccharides [104]. In these cells, chromatin memory depends on the activity of the Toll-like receptor (TLR; signal-dependent TF) which acts as a molecular transducer of environmental information, and the TF CCAAT/enhancer-binding protein beta (CEBPb; activity-dependent TF) induced by TLR and that initiate changes in chromatin accessibility [104]. CEBPb is expressed only transiently and plays a role in the initial establishment of chromatin states but not in their maintenance. The enrichment for binding motifs for the AP-1 family of TFs and constitutive TFs such as RUNX.1 and PU.1 in regions with chromatin memory in hematopoietic stem cells [104] suggests that some of these TFs may be involved in the maintenance of chromatin states similarly to that observed in epidermal stem cells.

### Histone modifications and variants

Genomic regions with chromatin memory in brain cells are also differentially enriched in histone post-translational modifications such as H3K4me1, H3K27ac, H3K79me2/3 among others [10, 12]. This suggests that histone modifications may be implicated in the establishment and maintenance of chromatin memory. Mechanistically, owing to their electrostatic properties, such modifications can help maintain an open chromatin configuration and favor permissive states for the binding of TFs and other regulatory proteins. In addition, TFs can recruit histone modifiers such as “writers,” “readers,” and “erasers” that catalyze the modification of histones in nearby nucleosomes [88, 89]. Since “writers” also possess domains that can bind to histone marks, they can reinforce histone modifications by positive feedback loops, and help maintain or even propagate chromatin states in the absence of the initiating signal [105, 106]. For instance, bromodomain proteins can recognize H3K27ac and promote the deposition of H3K4me3, reinforcing a configuration to chromatin favorable to TF binding [106]. However, recent evidence challenges a direct role of histone modifications in gene regulation and rather suggests that they are the bi-product of transcription [107–111]. For instance, in yeast, the presence of RNAPII at transcriptionally active sites can promote the recruitment and activity of histone acetyltransferases and favor histone acetylation [107]. Likewise in mammalian cells, transcription is required for the deposition of the active histone modifications H3K4me3 and H3K27ac and for the maintenance of H3K27me3, suggesting that histone marks reflect rather than induce active transcription patterns in cells [110]. Indeed conversely, H3K27 acetylation is dispensable for binding of the transcriptional machinery to chromatin in mESCs and for transcriptional activation during early differentiation [111]. A recent study also showed that gene transcription is not compromised during mESCs differentiation by the absence of H3K4me1 [108]. These observations suggest that histone modifications at loci displaying chromatin memory may be the by-product of RNA Pol II and TF occupancy, rather than be actively promoting their recruitment and the maintenance of a chromatin accessible state.

An alternative scenario in which histone modifications may directly influence chromatin memory is via changes in 3D genome organization. Such changes may affect the segregation of genomic regions carrying the modifications into separate compartments (A or B) leading to their isolation and stabilization within the nucleus [112]. Such segregation is favored by the presence of histones modifying enzymes such as methyltransferases and acetylases whose activity can influence the formation of genomic compartments by phase separation [112–114]. Indeed, the persistent decrease in H3K79me1/2 observed in the adult NAc after early life stress may involve chromatin segregation induced by histone modifying enzymes since it is associated with lower expression of Dot1l and Kdm2b, H3K79 methyltransferase and demethylase [12]. Other histone modifications may also be involved, for instance H4K5ac that is persistently increased by early life stress in NAc [12] and during fear memory formation in the hippocampus [115]. These results suggest that, at least in the context of early life stress, global changes in histone modifications may influence the establishment and maintenance of chromatin memory by being re-localized, stabilized, and protected in discrete regions of the nucleus. Notably, fear conditioning induces a long-term reconfiguration of the genome with many regions with chromatin marks changing compartment status [10]. However, whether this is driven by global changes in histone modifications is unknown.

Another mechanism that contributes to the maintenance of chromatin states is the replacement of histone variants. H2A.Z is one of the variants known to be reduced by chronic cocaine administration in the rodent brain and that remains low for up to 30 days after drug withdrawal [11]. H2A.Z is involved in nuclear functions, including RNA polymerase II pausing and enhancer activation during transcription [116, 117]. It is known to restrict the binding of AP-1 family TFs. In human cells, its depletion was shown to increase chromatin accessibility at AP-1 sites, likely reflecting the binding of TFs. This is consistent with the persistent gain in chromatin accessibility at AP-1-binding sites observed upon chronic cocaine that might have previously been occupied by H2A.Z. In particular, cocaine administration is accompanied by the transcriptional downregulation of *H2afz* and enhanced association of the H2A.Z chaperone ANP32E to chromatin [11]. Interestingly, *H2afz* gene itself remains significantly downregulated after chronic cocaine and during withdrawal, H2A.Z incorporation is reduced at promoter regions and in NAc-specific enhancers of genes such as *FosB* [11]. However, how H2A.Z occupancy correlates with lasting changes in chromatin accessibility during withdrawal remains not well understood. Since H2A.Z loss can lead to de novo recruitment of AP-1 [11], it may cause passive recruitment of other TFs that prime regulatory elements for future activation. This may explain the emergent transcriptional activation observed after withdrawal. Interestingly, the histone chaperone ANP32E specifically removes H2A.Z from chromatin and the level of ANP32E occupancy at the chromatin increases during withdrawal^1165,97^. This suggests that the reduced genomic binding of H2A.Z during withdrawal might result from lower *H2afz* transcription and increased H2A.Z removal, potentially affecting chromatin accessibility genome-wide.

### Additional factors influencing the establishment of chromatin memory

The establishment and maintenance of chromatin states induced by exposure are largely influenced by the developmental time window and duration of exposure. While long-lasting changes in chromatin structure can be potentially established any time during development, the molecular mechanisms allowing their stabilization and their cellular and functional consequences on the brain largely depend on the time of exposure. In particular, postnatal stages of brain development are periods of increased susceptibility because NSCs proliferate and differentiate into post-mitotic neurons that no longer divide nor undergo molecular “renewal” by replication. This means that epigenetic modifications acquired at that time may remain associated to the genome and constitute a form of chromatin memory in newborn neurons [65, 102]. Further, epigenetic states such as DNA methylation co-occurring with gene transcription may also be established in young neurons and prime chromatin until adulthood and influence future transcriptional dynamics [65]. Finally, progressive transcriptional repression of important epigenetic modifiers such as DNMT3A [118] and TFs [119] occurring during the first two weeks of postnatal development may also provide a memory of chromatin states. These processes may not operate in adult stages, and until now it is unclear if age plays a role in the acquisition of lasting changes in chromatin landscape.

## Functional consequences of lasting changes in chromatin in the brain

**Fig. 2:**
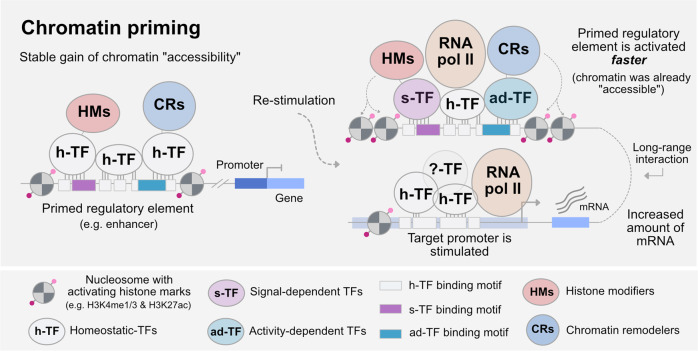
Model of chromatin priming and its functional consequence for gene expression. Priming of regulatory elements by TFs constitutes a form of chromatin memory for the control of stimulus-dependent gene expression (inspired by findings reported in [10, 101]). Left: primed regulatory element carrying activating histone modifications have a persistent increase in chromatin accessibility and a gain in h-TF, HMs and CRs as a result of previous activity (see Fig. 1, right). Upon re-stimulation, such as neuronal activation or inflammation, s-TF and ad-TF can be recruited to the primed regions at a faster rate and likely bind with stronger affinity to their target binding sites. A subset of h-TF could also remain associated [101]. The recruitment of s-TF leads to the activation of the primed regulatory element, an enhancer in this case, via recruitment of RNA pol II. The now activated regulatory element can engage in canonical or novel long-range interactions with the promoter of target genes [10] resulting in increased transcription [10, 101]. H3K4me1 and H3K27ac indicated in the figure have been associated with priming events in the brain [10, 91] but other histone modifications may contribute to the process. Also, while histone modifications have been associated with priming events, their functional contribution to gene expression has been recently challenged (see main text). The example provided here does not exclude additional functional consequences of chromatin memory in the brain.

In brain cells, changes in chromatin accessibility have been associated with differential gene expression. Exposure of NSCs to midazolam results in transcriptional upregulation of quiescence genes which correlates with increased chromatin accessibility at the promoter of these genes that persists in adult cells [90]. However, after neuronal activation, the majority of genes return to baseline although many genomic regions retain higher chromatin accessibility [10, 13, 74]. The apparent dissociation between lasting changes in chromatin accessibility at regulatory elements and minimal effects on transcription suggest that persist chromatin accessibility is a form of chromatin priming. Upon subsequent stimulation, primed regions can favor transcription by increasing transcriptional output, accelerating transcriptional activation or contributing to the emergence of new transcriptional programs (Fig. 2). For example, in the context of memory, neurons re-activated during recall have a different transcriptional program than neurons activated during memory formation [10]. The specific transcriptional program during recall correlates with a lasting increase in chromatin accessibility at enhancers and novel long-range interactions between enhancers and promoters [10]. Morphologically, re-activated neurons have more mushroom and thin spines, possibly as a consequence of transcriptional or chromatin changes of genes involved in spine formation. These results suggest that chromatin priming may drive differential transcriptional program after re-activation in part via the establishment of novel long-range interactions.

Pathological neuronal activation such as during epileptic seizure (or status epilepticus) can also result in long-lasting changes in chromatin accessibility at key regulatory elements and potentially affect transcriptional programs. Mice subjected to seizure by kainic acid have memory deficits and blunted *c-Fos* expression associated with a lasting gain in chromatin accessibility at *Jdp2*, a gene located downstream of *c-Fos* that encodes an AP-1 repressor [13]. Changes in brain chromatin associated with pathological immune conditions and involving differences in H3K4me1 and H3K27ac at enhancer elements have also been linked to neurodegenerative diseases. In a mouse model of Alzheimer’s disease, such immune memory modulates the deposition of amyloid plaques and reduces neuronal damage and microglia activation after brain ischemia [13]. Overall, changes in chromatin can influence transcriptional programs in basal, stimulated and pathological conditions and can affect cell physiology in the brain, impairing cognitive abilities and promoting neuropathology.

## Conclusions and perspectives

Experiences are major determinants of brain functions that shape developmental and molecular trajectories of brain cells and have lasting effects influencing neural responses and behavior in adulthood. While synaptic processes are classically thought to be the main site for neural stability and plasticity, new findings indicate that chromatin also has these properties. Its composition, structure and 3D organization within the cell nucleus can contribute to transcriptional programs activated by experience and act as mediator of information assimilation in the genome in the form of chromatin memory.

Mechanistically, the establishment and maintenance of chromatin memory rely on the activity of TFs that bind to genomic sites upon stimulation, and on the stabilization of activated chromatin states. Such mechanisms have been implicated in inflammatory memory in immune cells and epithelial stem cells, and we postulate that they are also at play in brain cells. We propose that experiences can lead to persistent changes in chromatin structure and organization via differential activity of AP-1 TFs. Since these TFs have been implicated in signal-induced long-range interactions between regulatory elements [120], they may as well cause persistent 3D genome reorganization. The systematic characterization of chromatin accessibility and genome 3D organization coupled with genome-wide profiling of AP-1 TFs binding after exposure should identify regulatory responses mediating the establishment and maintenance of chromatin memory in brain cells (see models in Figs. 1 and 2).

While important progress has been made in the understanding of chromatin regulation in the brain, several major questions about the mechanisms of chromatin memory and their implication in brain functions and diseases remain open and need experimental work to be addressed. In particular, studies on the formation of chromatin memory in brain cells with a better temporal resolution (from minutes/hours to days or months after exposure) coupled with genome-wide characterization of binding profiles of AP-1 TFs, histone modifications and cell-type specific and constitutive candidate TFs are needed. Such experiments should ideally be conducted in vivo but also in vitro using primary cultures, differentiated ESCs or even brain organoids, and analysis may focus on activated cells to maximize the detection of effects. Experiments designed to identify and characterize the temporal dynamics of chromatin memory formation and maintenance should also help clarify the stability of changes. It is also still unclear how long after exposure changes in chromatin can be considered stable or still a remnant of regulatory dynamics operating after stimulation. Further, studies characterizing the acquisition of chromatin memory in brain cells in response to the same stimulus but at different period of life (early postnatal, adolescence, adulthood, or aging) should inform on the effect that time of exposure can have on the vulnerability or resilience of the genome to lasting changes in chromatin and whether this correlates with cognitive and behavioral alterations.

The available evidence at the moment suggests that chromatin memory is a more widespread phenomenon than previously anticipated. Therefore, its study in the context of brain functions should not be limited to neurons, unlike done so far, but should also include glial cells such as microglia, and even epithelial cells, since environmental exposures are likely to affect all cell types in the brain. Further, multiple brain regions in addition to classical hippocampus, such as the medial pre-frontal cortex should be examined. Critically, the functional consequences of chromatin memory in the brain remain to be clarified but likely involve transcriptional programs that affect brain cells physiology and therefore, behavior and cognition. The manipulation of regulatory sequences with chromatin memory in vitro or in vivo could inform on their contribution to transcriptional programs after experience and to changes in behavior and cognition.

Finally, while accumulating evidence strongly supports the existence of chromatin memory in brain cells, this evidence is mostly derived from animal models, rodents in particular. Therefore, there is also a need to address the existence of chromatin memory, its mechanisms and potential functional implications in human brain cells. In this regard, the use of neurons and glia derived from induced pluripotent stem cells (iPSCs) in culture or in the form or organoids coupled with different types of exposure should provide valuable models to characterize chromatin memory in human brain cells. We anticipate that mechanisms of chromatin memory identified in rodents also operate in human cells, however, direct experimental evidence is needed to support such claim. We postulate that lasting changes in chromatin structure and organization in human cells, acquired during our life time, might not just influence cognition and behavior but also the likelihood for the development of psychiatric and mood disorders as well as the origin and evolution of neurodegenerative diseases.

## Data Availability

Availability of data described in the review is described in relevant research manuscripts (see bibliography).
